# Intensive Surveillance and Aggressive Multimodal Treatment for Liver Metastases From Uveal Melanoma

**DOI:** 10.1097/AS9.0000000000000620

**Published:** 2025-10-08

**Authors:** Hemant M. Kocher, Akil Gani, Ziya O. Belibagli, Adithi Shankar, Amina Saad, Karen Mawire, Amr Wassef, Jo-Anne Chin Aleong, Michael Sheaff, Gordon Stamp, Mandeep S. Sagoo, Peter W. Szlosarek

**Affiliations:** From the * Centre for Tumour Biology, Barts Cancer Institute – a CRUK Centre of Excellence, Queen Mary University of London, London, UK; † Barts and the London HPB Centre; ‡ NIHR Barts Biomedical Research Centre, Queen Mary University of London, London, UK; § The London Clinic, London, UK; ‖ NIHR Biomedical Research Centre for Ophthalmology at Moorfields Eye Hospital and UCL Institute of Ophthalmology, London, UK; ¶ Ocular Oncology Service, Moorfields Eye Hospital NHS Foundation Trust, London, UK; # Department of Cellular Pathology, The Royal London Hospital, Barts Health NHS Trust, London, E1 1BB. UK; ** List of Contributors is available in ACKNOWLEDGMENTS section.; (The Royal London Hospital and The London Clinic); (The Royal London Hospital); (The Royal London Hospital); (The Royal London Hospital); (The London Clinic); (The London Clinic); (The Royal London Hospital and The London Clinic); (The Royal London Hospital); (The Royal London Hospital); (The Royal London Hospital); (The Royal London Hospital); (The London Clinic); (The London Clinic); (St Bartholomew’s Hospital and The London Clinic); (St Bartholomew’s Hospital); (St Bartholomew’s Hospital); (Moorfield’s Eye Hospital and The London Clinic); (Moorfield’s Eye Hospital); (Moorfield’s Eye Hospital); (posthumous); (The London Clinic); (The Royal London Hospital and The London Clinic); (The Royal London Hospital); (The Royal London Hospital); (The Royal London Hospital); (The Royal London Hospital); (The Royal London Hospital)

**Keywords:** uveal, laparoscopy, liver resection, liver ablation, metastasis

## Abstract

**Objective:**

We evaluated a cohort of patients with liver metastasis from uveal melanoma (LMUM) to assess the benefit of intensive surveillance and multimodal treatment on overall survival.

**Background:**

LMUM is typically associated with a poor prognosis.

**Patients and methods:**

This two-center retrospective cohort study from January 2010 to December 2024 included 58 patients with LMUM deemed to be oligometastatic and referred for surgical management. Overall survival after treatment of LMUM and primary uveal melanoma was determined using Kaplan–Meier methods and the Cox proportional hazards method.

**Results:**

Fifty-eight patients [performance status (PS): PS1 = 11, PS0 = 47] with oligometastatic LMUM were screened to stratify patients with multifocal disease not undergoing liver surgical/ablative treatment (Group A, n = 27) and those with oligometastatic liver disease having liver resection/ablation (Group B, n = 31) along with systemic treatment as per patient/physician choice. Patients in Group B had longer liver-specific overall survival [Group B: OS = 45.1 (95% confidence interval (CI) = 33.5–not reached] months; Group A, median 18.6 (95% CI = 13.8–23.8) months; *P* < 0.0001, log-rank (Mantel-Cox) test, hazard ratio (HR): 0.13, 95% CI = 0.06–0.28) and better overall survival from initial treatment for primary uveal melanoma [Group B 14.1 (95% CI = 8.2–20.8) years vs Group A 3.6 (95% CI = 2.5–5.5) years; *P* < 0.0001, HR: 0.24, (95% CI = 0.11–0.50)].

**Conclusions:**

Intensive surveillance for early diagnosis of oligometastatic LMUM and its relapse along with surgical resection/ablation and systemic treatment facilitates long-term remission. This retrospective case series requires prospective validation in a multicenter cohort study.

## INTRODUCTION

Uveal melanoma (UM), arising from either ciliary body (~10%), iris (<10%), or choroid (>80%), is a rare condition representing 3% to 5% of all melanomas, occurring at 5 cases per million in the Caucasian population.^1,2^ Its primary treatment includes brachytherapy, proton beam therapy, or enucleation, whereas surgical local resection, thermotherapy, and photodynamic therapy are rarely used.^3^ Surveillance with liver-directed imaging by ultrasound [or magnetic resonance imaging (MRI)] is recommended, since around 50% of all patients will develop metastases, with the liver being the preferred organ for dissemination (80–90%).^4^

Median survival after detection of liver metastasis from uveal melanoma (LMUM) is 10 to 22 months, with an urgent need to develop multimodal treatment options.^5–12^ Due to the rarity of LMUM, evidence from randomized clinical trials is limited, but treatment options include immunotherapy, ablation, surgical resection, and trans-arterial targeted treatments, often extrapolated from nonuveal solid cancers frequently metastasizing to the liver.^2,11–20^ The number and size of metastases, along with age, are key prognostic variables.^17,21,22^ Surgical resection for LMUM is favored by many specialist centers in fit patients with oligometastatic spread,^23–29^ either alone or in combination with ablation.^30^ Laparoscopy, to stratify selection for surgical resection/ablation, may aid detection of further metastasis below the threshold of detection for MRI and positron emission tomography-computed tomography (PET-CT), since melanoma metastases frequently are distinctively pigmented and predominantly on the liver surface.^26,27^

The aim of this 2-center study is to report utility of diagnostic laparoscopy, long-term survival outcome for patients undergoing multimodal treatment for LMUM, and determine predictive factors for survival.

## METHODS

### Study Design

After treatment of primary UM, patients were followed up locally with regular liver ultrasound (or MRI scan) and ophthalmic examination every 6 months. If metastases were suspected, a CT scan and/or MRI were performed to assess disease burden, extrahepatic metastases, and resectability, and complemented with PET-CT if required. Patients with suspected oligometastatic liver-only disease were discussed in a hepato-pancreatico-biliary (HPB) and ocular oncology multidisciplinary team (MDT) to devise a patient-specific plan based on current applicable guidance.^31^ Liver resection (and/or ablation), subject to laparoscopy, was suggested for patients with no extrahepatic disease, liver imaging indicating a feasible curative resection, and no prohibiting comorbidities. Eligible patients underwent diagnostic laparoscopy, thoroughly exploring the entire abdominal cavity with the liver evaluated by inspection and intraoperative ultrasound, as well as biopsy (indications: uncertain diagnosis, unresectable disease). If feasible laparoscopically, liver metastasectomy was performed during the same procedure. Most often, a subsequent laparotomy was performed for the liver resection. Patients on treatment were subsequently followed up with 3 to 6 monthly MRI (and/or PET-CT) to detect early relapse, which could be acted upon immediately after discussion at HPB MDT.

### Patients and Assessments

From January 2010 to December 2024, all consecutive patients with suspected LMUM discussed at HPB MDT were identified from our prospectively maintained surgical database (Barts Health National Health Service (NHS) Trust, Institutional approval 13499, August 2024; Moorfields Eye Hospital NHS Foundation Trust, Institutional approval 1631, December 2024). Data were collected on demographics, characteristics of primary UM treatment, age at diagnosis of UM and its metastases, and interval between primary tumour treatment and diagnosis of liver metastasis. For the index metastatic liver event, the following items were recorded: number, location of metastases on imaging and diagnostic laparoscopy, presence of miliary disease on diagnostic laparoscopy, extrahepatic disease during surveillance period, recurrence of metastases, other treatments administered, and date of last follow-up or death. For primary UM, the nature of the lesion (histology and size) as well as treatment modalities were retrospectively obtained. Data were censored on December 30, 2024.

### Statistical Analysis

Overall survival (OS) from primary disease was calculated as the time from date of primary disease treatment to date of last follow-up (censored) or death. Liver-specific OS was calculated from the date of index liver resection/ablation to the date of last follow-up (censored) or death. Descriptive data analysis was performed using Microsoft Excel (version 2020; Microsoft Corporation, WA). Survival outcomes were calculated using the Kaplan–Meier survival curves (log-rank test) and multivariate analysis with Cox proportional hazards models using GraphPad Prism 10.1.2 (GraphPad Software, Inc., San Diego, CA). Statistical tests with a *P* value <0.05 were considered significant.

### Data Availability

De-identified summary patient data are available in Tables 1 and 2 and Supplementary data https://links.lww.com/AOSO/A543. Further data is available from the corresponding author upon reasonable request.

**TABLE 1.** Patient Characteristics in Group A & B and For the Whole Cohort of Liver Metastases from Uveal Melanoma

	Overall Cohort	Group A	Group B	*P* value	Statistical Test
Male sex—no. (%)	25 (43.1)	12 (44.4)	13 (41.9)	0.35	Chi-square
LMUM presentation characteristics					
Median age (IQR)—yr LMUM diagnosis	65 (57–75)	68 (59–79)	63 (56–71)	0.12	Mann–Whitney
ECOG performance-status score—n				0.06	Chi-square
0	47	19	28		
1	11	8	3		
Median LDH (IQR)	241 (194–401)	393 (209–969)	217 (158–361)	0.41	Mann–Whitney
LDH unavailable	49	22	27		
Median alkaline phosphatase (IQR)	86 (58–107)	85 (55–127)	88 (58–107)	0.97	Mann–Whitney
ALP unavailable	18	10	8		
Largest metastatic lesion—n				0.35	Chi-square
≤3.0 cm, stage M1a	48	21	27		
>3.1, stage M1b/c	10	6	4		
Location of metastasis—n				**<0.0001**	Chi-square
Unilobar—n	33	7	26		
Bilobar—n	25	20	5		
Primary UM disease characteristics					
Median age (IQR)—yr primary UM diagnosis	59 (53–72)	65 (55–77)	55 (50–66)	**0.025**	Mann–Whitney
Median interval between UM and LMUM diagnosis (IQR)—yr	2.7 (1.4–5)	1.9 (1.1–2.8)	3.7 (2.6–5.9)	**0.002**	Mann–Whitney
Histological subtype				0.62	Chi-square
Epithelioid	9	6	3		
Spindle	5	2	3		
Mixed	5	2	3		
Histological subtype unavailable	39	17	22		
Median tumor (ultrasound) thickness (IQR) mm		6.3 (3.2–9.3)	6.6 (3.8–8.0)	0.98	Mann–Whitney
Tumor (ultrasound) thickness unavailable (n)	14	0	14		
Median tumor (ultrasound) diameter mm (IQR)		13.3 (11.5–19)	12 (10.7–13.5)	0.06	Mann–Whitney
Tumor diameter unavailable (n)	14	0	14		
Primary initial treatment				0.43	Chi-square
Plaque brachytherapy (n)	20	11	9		
Proton beam therapy (n)	10	3	7		
Enucleation (n)	28	13	15		
Primary repeat treatment					
Proton beam therapy (n)	2	0	2	0.33	Chi-square
External beam radiotherapy (n)	3	1	2		
Enucleation (n)	7	4	3		

Statistical test for univariate comparison between Group A and B. Significant P value is highlighted in bold.

**TABLE 2.** Multivariable Analysis for Overall Survival After Treatment for LMUM and UM

Cox Proportional Hazard Model: Hazard Ratio (95%CI)	Survival After LMUM	Survival After UM
Group A vs B	0.14 (0.04–0.45)	0.21 (0.06–0.69)
Age at UM Diagnosis	1.01 (0.99–1.05)	1.02 (0.99–1.05)
Interval Between UM and LMUM	0.99 (0.99–1.00)	0.99 (0.99–1.00)
Unilobar/Bilobar Metastases (Bilobar)	0.67 (0.25–1.94)	1.03 (0.37–3.05)

## RESULTS

### Patient Population

**FIGURE 1.**
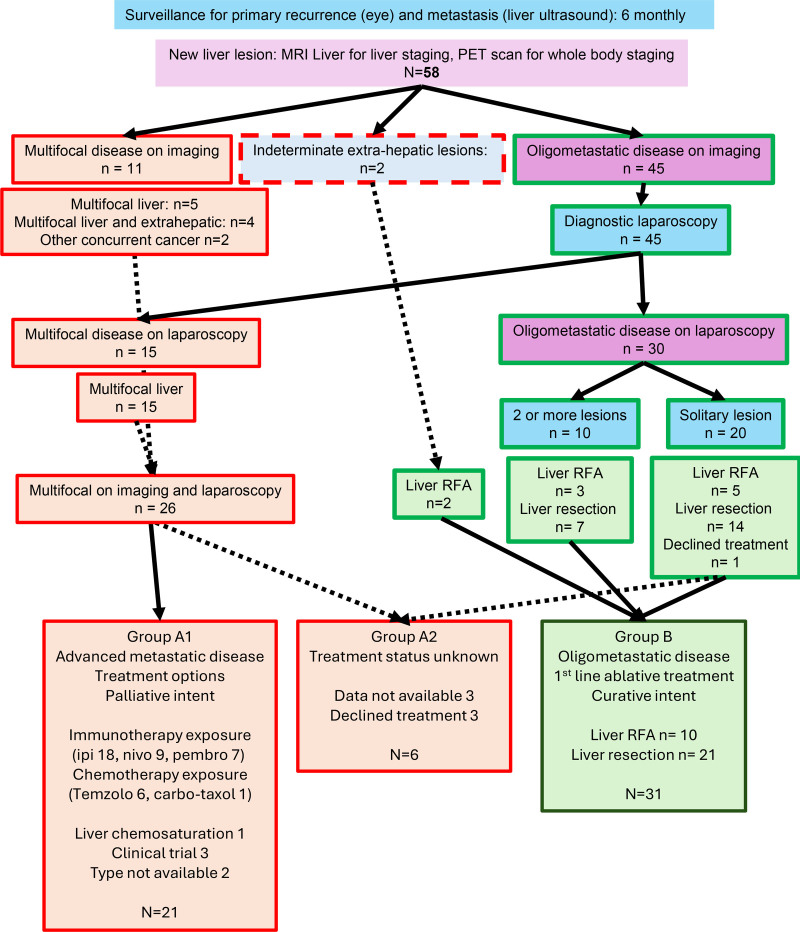
Consort diagram of screening for oligometastatic disease after referral for surgical treatment of liver metastases from uveal melanoma. Stratification of patients into those with multifocal disease [Group A (sub-groups A1/A2)] as well as those with oligo-metastatic disease undergoing surgical resection or ablation of liver lesion (Group B) based on information from imaging and diagnostic laparoscopy. Carbo-Taxol indicates carboplatin and paclitaxel; Ipi, ipilimumab; Nivo, nivolumab; Pembro, pembrolizumab; RFA, radiofrequency ablation; Temzolo, temozolomide.

All consecutive patients referred to HPB MDT at either institution for consideration of liver resection or ablation after suspected LMUM were included in this cohort analysis (Fig. 1, Table 1). Patients were offered treatment options based on MDT decision and patient/physician choice. Eleven of the 58 (19%) patients had multifocal disease or other concurrent cancer diagnosed on further imaging assessments (dedicated MRI liver, CT, and/or PET-CT scan, as part of our routine protocol). Two patients with an indeterminate lung (n = 1) and spleen (n = 1) lesion had liver ablation. Forty-five patients with presumed oligometastatic liver-only disease were further assessed with diagnostic laparoscopy, to reveal a further 15 (33%) to have multifocal hepatic metastases, precluding liver resection/ablation, and 1 patient declined further treatment. Thus, 27 patients (46.5%) did not undergo liver resection/ablation, mostly due to multifocal disease (Group A). Eleven of these 27 patients (41%) went on to have extrahepatic progression on further assessments and multiple lines of systemic treatment without surgery or ablation (Supplementary Figure 1, https://links.lww.com/AOSO/A543).

### Surgical Resection

**FIGURE 2.**
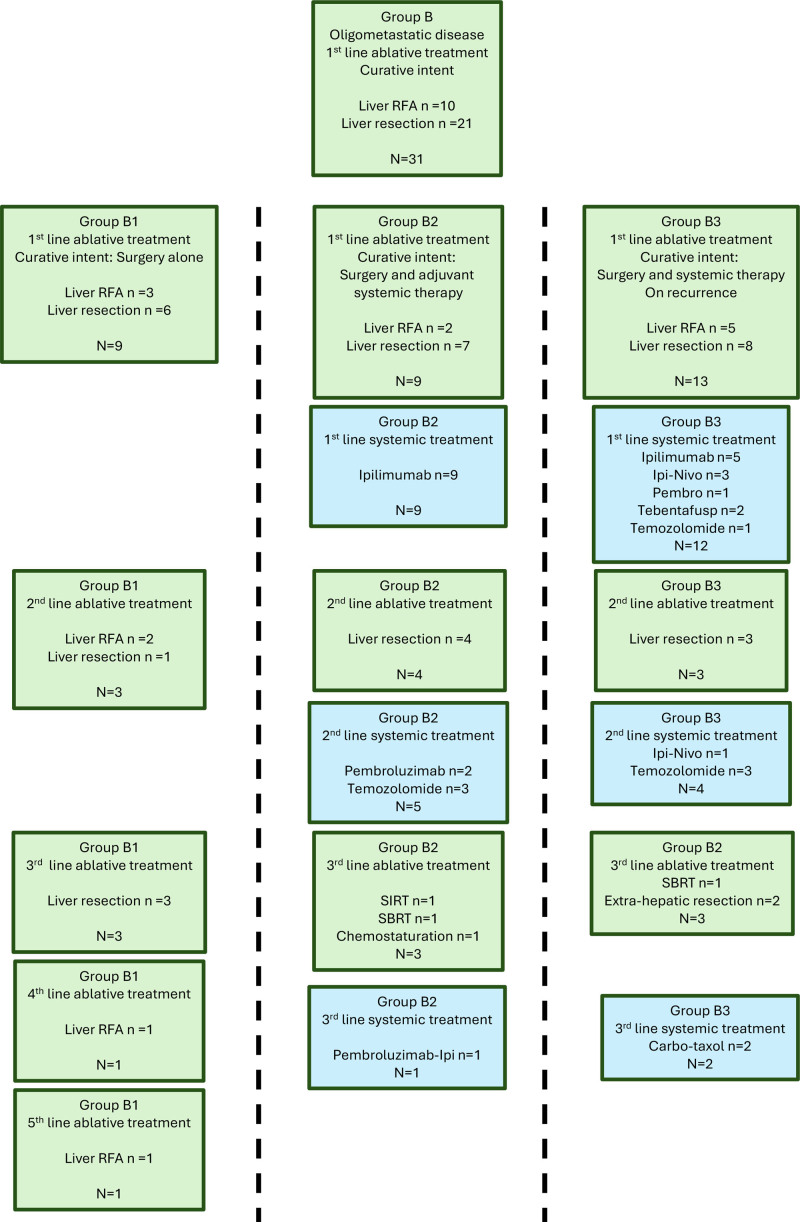
Surgical and systemic treatment options for oligometastatic liver metastasis from uveal melanoma. Types of treatment for patients with oligo-metastatic disease undergoing surgical resection or ablation of liver lesion (Group B) based on those electing not to have adjuvant systemic treatment (Group B1), those electing to have adjuvant systemic treatment (Group B2), and those having systemic treatment on discovery of relapse (Group B3). Carbo-Taxol indicates carboplatin and paclitaxel; Ipi, ipilimumab; Nivo, nivolumab; Pembro, pembrolizumab; RFA, radiofrequency ablation; SBRT, stereotactic body radiation therapy; SIRT, selective internal radiation therapy; Temzolo, temozolomide.

Twenty-one of 58 patients with LMUM had liver resection as a primary modality, with the majority (18/21) achieving an R0 resection margin, and a further 10 had liver ablation (Group B), with the choice determined during a shared decision-making process between physician and patient after HPB MDT discussion. None of these patients had any systemic treatment before liver resection or ablation. All patients were offered adjuvant immunotherapy and were surveyed intensely (MRI Liver 3–6 monthly with FDG-PET as indicated). While some patients opted never to have immunotherapy (n = 9, Group B1), some had adjuvant treatment (n = 9, Group B2), the remaining had immunotherapy after recurrence was detected (n = 13, Group B3). However, 28 of 31 patients (90%) had liver relapse as primary site of recurrence, and 10 (32%) patients went on to have extrahepatic progression. Multimodal treatment was offered for all patients (Fig. 2). Ten patients went on to have a second liver resection/ablation, and 5 patients had a third liver resection/ablation for oligometastatic liver progression. Two patients had oligometastatic extrahepatic progression resected. Multiple lines of immunotherapy/chemotherapy were offered upon progression. Retrospective review of treatment modalities for primary UM showed a similar distribution for initial and repeat treatment for primary UM across Group A and B (Table 1, Supplementary Figure 2, https://links.lww.com/AOSO/A543).

### Survival Analysis

**FIGURE 3.**
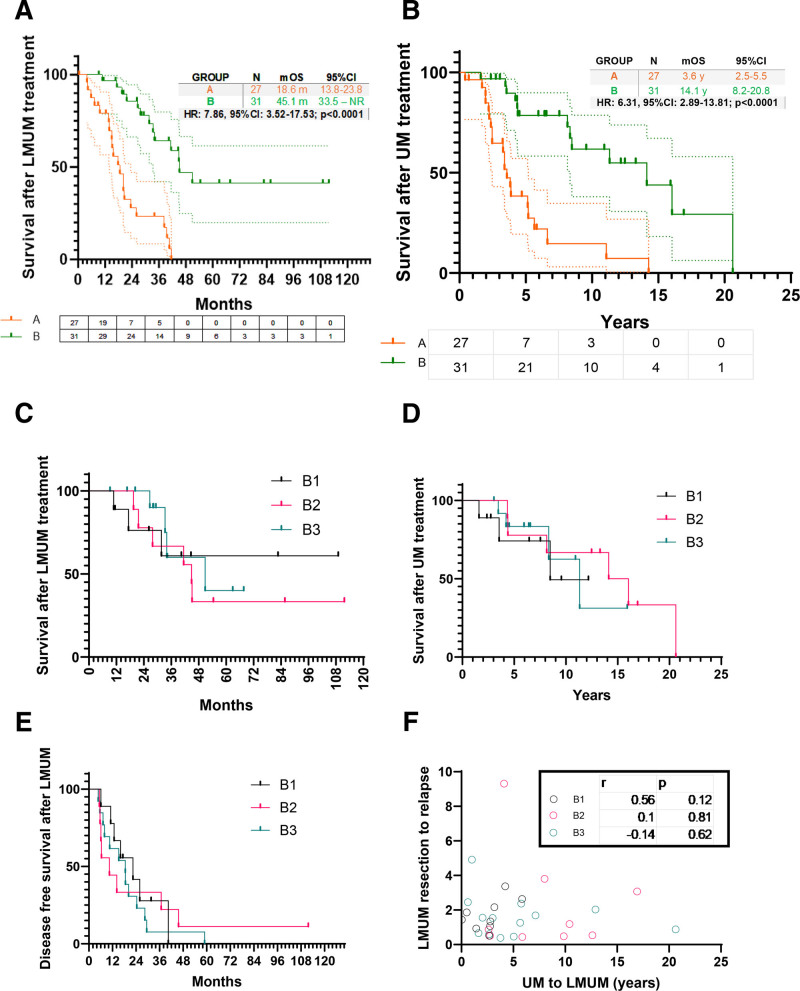
Survival features after treatment of liver metastases from uveal melanoma. A, Overall survival from the treatment of LMUM [Group A (orange) vs Group B (green)]. Numbers at risk are shown along X-axis. 95% CI shown for survival probability for both groups. B, Overall survival from the treatment of primary uveal melanoma (UM) [Group A (orange) vs Group B (green)]. Numbers at risk are shown along X-axis. 95% CI shown for survival probability for both groups. C, Overall survival from the treatment of LMUM [Groups B1 (black), B2 (pink), B3 cyan)]. D, Overall survival from the treatment of primary UM [Groups B1 (black), B2 (pink), B3 cyan)]. E, Disease-free survival after treatment of LMUM [Groups B1 (black), B2 (pink), B3 cyan)]. F. Correlation between disease-free survival after resection/ ablation of LMUM and interval between treatment of UM and LMUM [Groups B1 (black), B2 (pink), B3 cyan)]. A–E, log-rank (Mantel-Cox) test. F, Spearman's correlation test.

Patients with oligometastatic disease undergoing liver resection/ablation as primary modality of treatment (Group B) had significantly longer OS [*P* < 0.0001, log-rank (Mantel-Cox) test, hazard ratio (HR): 0.13, 95% confidence interval (CI) = 0.06–0.28] after treatment of metastatic disease compared with multifocal disease treated with immunotherapy as primary modality (Group A), where all patients were treated with multiple lines of systemic treatment (Fig. 3A). This was also seen for OS from initial treatment for primary UM (Fig. 3B) [*P* < 0.0001, log-rank (Mantel-Cox) test, HR: 0.24, (95% CI = 0.11–0.50)]. While the age of diagnosis of LMUM was similar for both Groups A (multifocal metastasis, median 68 years) and B (oligometastatic disease, median 63 years) (2-tailed Mann–Whitney *U* test, *P* = 0.12, Table 1, Supplementary Figure 3A, https://links.lww.com/AOSO/A543), patients in group B were younger at time of diagnosis of primary UM [median 55 (B) versus median 65 years (A), two-tailed Mann–Whitney *U* test, *P* = 0.035, Table 1, Supplementary Figure 3B, https://links.lww.com/AOSO/A543) with a longer interval to metastatic progression (median 1373 days (B) versus median 690 days (A), two-tailed Mann–Whitney *U* test, *P* = 0.002, Table 1, Supplementary Figure 3C, https://links.lww.com/AOSO/A543). Nearly 39% of patients in Group B presented with LMUM beyond 5 years of diagnosis of primary UM (Fisher exact test, *P* < 0.0001), with a similar number in synchronous presentation (<1 year, n = 5 each group), and most presenting within the 1 to 5 year period after treatment for primary UM (Supplementary Figure 3C, https://links.lww.com/AOSO/A543).

Despite small numbers in subgroups for Group B, we carried out a subgroup analysis to show no differences in OS from diagnosis of primary UM or that of LMUM (Fig. 3C,D) or hepatic disease relapse (Figure 3E), with no differences in age at presentation of UM or LMUM (Supplementary Figure 3D–E, https://links.lww.com/AOSO/A543), but with a slightly longer interval between UM and LMUM presentation in Group B2 compared with group B1 (Supplementary Figure 3F, https://links.lww.com/AOSO/A543).^28^ Interestingly, the correlation between time interval to metastatic progression to time interval to metastatic relapse after resection/ ablation of LMUM was not observed in any of the subgroups (Fig. 3F).

### Univariate and Multivariate Analysis

On univariate analysis, parameters associated with prolonged OS after liver resection (liver-specific OS) and OS from primary disease treatment were patients with unilobar, oligometastatic disease intraoperatively/imaging, those who had an interval of more than 3 years between primary disease treatment and liver metastases diagnosis as well as those patients who underwent liver resection, with no prognostic impact from lactate dehydrogenase, alkaline phosphatase values at time of LMUM diagnosis or characteristics of primary tumor or its treatment (Tables 1 and 2). On multivariate analysis, OS from primary UM or LMUM treatment was ability to perform liver resection/ ablation (Table 2).

## DISCUSSION

This largest series of patients with LMUM from the United Kingdom emphasizes the vital role of diagnostic laparoscopy to rule out bilobar miliary disease before definitive liver surgery as advocated by the Uveal Melanoma UK National Guidelines (2015).^31^ We suggest the benefit of surgical resection in improving OS for LMUM, in highly selected individuals, to a median OS of 45.1 (95% CI = 31 months–not reached) months after diagnosis of LMUM.^23–25,27–29,32^ We confirm that oligometastatic disease and a long interval between primary UM and LMUM (>3 years) influence a good outcome.^7,24,28^ Most importantly, the multivariate analysis demonstrates aggressive surveillance leading to timely detection of oligometastatic relapse and its subsequent multimodal treatment, with surgical resection (liver and other sites) underpinning treatment options, prolongs survival beyond currently known and accepted standards for metastatic UM. In comparison to other more common cancers, such as colorectal cancer liver metastases, this case series is small and retrospective, limiting its generalisability. Ideally, a multi-center validation of this aggressive approach with prospective algorithms, as well as in-depth study of biological factors, could improve care for patients with LMUM.

For example, the largest series of patients with LMUM (n = 255/1991–2007) undergoing liver surgery with R0 resection (n = 76) or R1 resection (n = 22) or R2 resection (n = 157) suggested that absence of miliary disease, number of metastases resected (≤4), R0 margin, and >24-month interval from primary tumor diagnosis to liver metastases (‘lead time’) were prognostic determinants in multivariate analysis.^24^ Their median overall postoperative survival was 14 months but increased to 26 months when R0 resection was achieved in 76 patients, while the median OS was 37 months in isolated liver resection for LMUM.^28^ Interestingly, with selective use of laparoscopy (when more than 4 lesions were suspected on imaging) resulted in a large proportion (157/255 R2 and 22/255 R1) of incomplete resections. A smaller UK (Liverpool, n = 155, 2004–2012) study assessed 17 patients (88% R0 resection) undergoing liver resection with a median OS of 27 months.^27^ Of note, in these older series, patients would not have benefited from immunotherapy agents such as ipilimumab. Furthermore, surveillance or second resection/ablation was not commented upon in these older series. However, these results, along with this current series, confirm that diagnostic laparoscopy is a vital tool in the evaluation of patients with LMUM being considered for R0 liver resection. Laparoscopy may be difficult in patients with previous abdominal operations, resulting in perihepatic adhesions precluding assessment. Meta-analyses have suggested that raised lactate dehydrogenase and serum alkaline phosphatase may prognosticate for OS after detection of liver metastasis, while age, gender, and diameter of the largest metastases were significant only on univariate analysis in patients not undergoing surgery but being exposed to chemo-/immune-therapy.^11^ Prognostic models such as Liverpool Uveal Melanoma Prognosticator Online (LUMPO III),^3^ which estimates both metastatic and nonmetastatic mortality according to anatomical, pathological, and genetic factors, as well as patient age and gender, is evolving in its ability to predict survival after primary UM diagnosis but not after development of LMUM. Similarly, Helsinki University Hospital Working Formulation tries to predict survival after metastatic development but has been found not to be prognostic when surgical resection of LMUM has been carried out.^33^

The 45-month median OS of patients with LMUM in our cohort—largely exposed to postoperative single-agent ipilimumab (14/30) or with other agents (7/30)—is superior to the recent immunotherapy studies in this disease achieved with dual agent immune checkpoint blockade [19.1 months (95% CI = 9.6–NR), n = 35]^19^ and tebentafusp in HLA-A*02:01 individuals [21.6 months (95% CI = 19.0–24.3), n = 252].^12^ Moreover, the 3-year survival in our Group B cohort (60%) is encouraging compared with recent secondary analysis of a phase III clinical trial exploring the clinical benefit of tebentafusp,^12^ where 27% of patients are alive, and emphasizes the important role of loco-regional surgical-based therapy. Indeed, even at 5 years (46%), our results compare favorably to a recent analysis of noncutaneous melanoma treated with local therapies and immune checkpoint blockade to show 5-year survival of 22% for UM-specific disease.^34^ Thus, the present study adds further weight to the combined role of loco-regional treatment, such as surgical resection/ablation and systemic immunotherapy, in extending long-term survivals. Furthermore, we emphasize the added value of intensive surveillance and proactive further surgical/ablative therapies and/or chemo or immunotherapy. We demonstrate a lack of correlation between time interval to metastatic progression and to time interval to metastatic relapse after resection/ablation of LMUM. This would need to be validated in a larger prospective series.

Many prognostic parameters after LMUM have been described in the literature.^29^ Prolonged survival is associated with increased ‘lead time,’ where some studies have described this as >24 months and some as ≥45 months. Similarly, the number of metastatic lesions also appears to play a crucial role in prognostication, with some studies describing patients having less than 10 metastases having improved survival, while others cite less than 4 metastases having good prognostication.^26,35^ Other parameters associated with improved survival described include age <70 years, absence of extrahepatic disease, and disomy 3 tumoral status, which is linked to reduced metastases and significantly slower metastatic disease progression.^36^ Interestingly, extrahepatic disease responds better to immunotherapy than LMUM.^19^ Genetic analysis (GNAQ, GNA11, BAP1, SF3B1 mutations; and chromosome 3 and 8 status are commonly studied) may also be prognostic after LMUM as they do after UM primary diagnosis^3^ but this analysis was not possible in our cohort.

With regard to treatment options, alongside surgery, radiofrequency ablation (RFA) has also been advocated to spare hepatic parenchyma, with some studies reporting similar survival times in groups having RFA ± liver surgery or liver surgery alone (27 months/28 months).^24^ Another study analyzed 14 patients with the disease, showing that patients with first recurrence can be treated with either RFA alone or RFA ± surgery and can have prolonged long-term survival (70% at 5 years).

In summary, the data presented from London, UK, highlight an important role for loco-regional surgical/ablative therapy in LMUM, providing the best survival outcomes reported to date in this disease subset. While case selection may be an important factor, our intensively managed surgical series of patients argues for further studies incorporating resection of oligometastatic disease and adjuvant immunotherapy in the treatment of LMUM.

## ACKNOWLEDGMENTS

Contribution from The London Clinic HPB MDT and Barts & the London HPB MDT and Ocular Oncology MDT at Moorfields Eye Hospital are as follows:

Data collection was done by Ziya O. Belibagli, Amina Saad, Adithi Shankar (The Royal London Hospital); Amr Wassef (Moorfields Eye Hospital); and Akil Gani, Karen Mawire (The London Clinic). Histopathology diagnosis and contribution was done by Jo-Anne Chin Aleong, Michael Sheaff (The Royal London Hospital); and Gordon Stamp (The London Clinic). Akil Gani led the analysis and first draft of the manuscript.

Interventional radiology procedures and contribution was by Ian Renfrew (The Royal London Hospital and The London Clinic); Deborah Low, Tim Fotheringham, Amr Elsaadany (The Royal London Hospital); and Robert Thomas, Steven Moser (The London Clinic).

Cross-sectional radiology procedures and contribution was by Khawaja Shahabuddin (The Royal London Hospital and The London Clinic); Mahrukh Qureshi, Niall Power, Marvin Daglish, Rohit Malliwal (The Royal London Hospital); and, Marc Pelling, Zahir Amin (The London Clinic).

Medical Oncology procedures and contribution was by Peter Szlosarek, (St Bartholomew’s Hospital and The London Clinic); and Sukaina Rashid, Shanthini Crusz (St Bartholomew’s Hospital).

Ocular Oncology procedures and contribution was by Mandeep Sagoo (Moorfield’s Eye Hospital and The London Clinic), Amit K Arora, Gordon Hay (Moorfield’s Eye Hospital); and Victoria Cohen (posthumous), Vasilios Papastefanou (The London Clinic), Surgical Oncology procedures and contribution Hemant Kocher (The Royal London Hospital and The London Clinic); and Ajit Abraham, Satyajit Bhattacharya, Vincent Yip, Deepak Hariharan, Sivasanker Masillamany (The Royal London Hospital).

## Supplementary Material


