# Supplementary figures and images for: Intensive Surveillance and Aggressive Multimodal Treatment for Liver Metastases From Uveal Melanoma
Source: Ann Surg Open. 2025 Oct 8;6(4):e620. doi: 10.1097/AS9.0000000000000620 (PMC12727333; doi:10.1097/AS9.0000000000000620)

Supplementary Figure 1

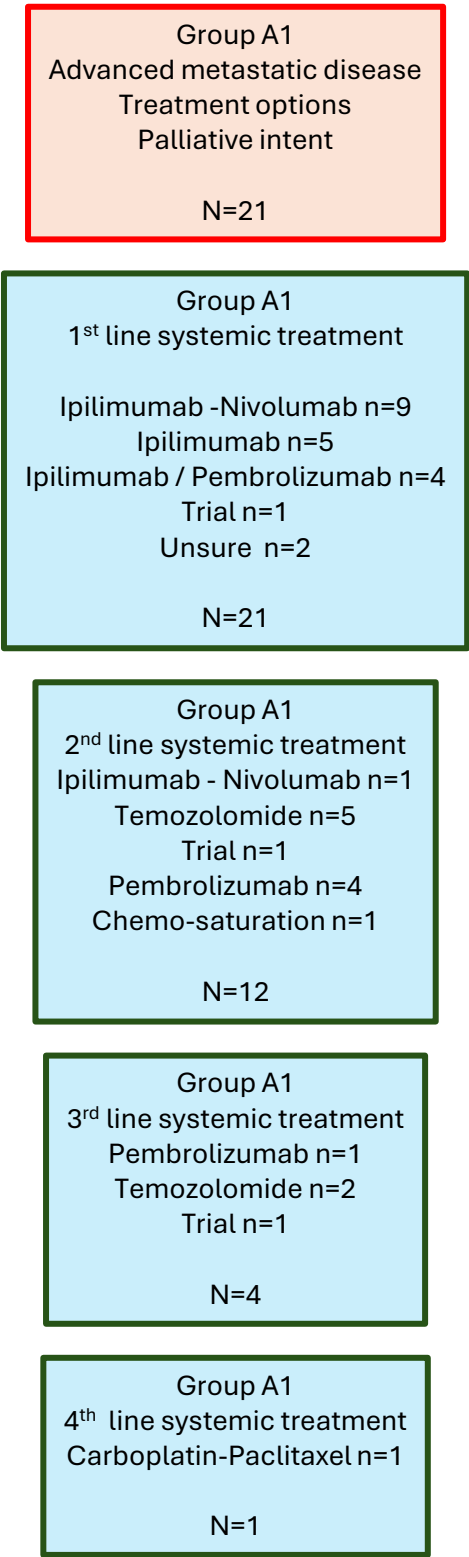

Supplementary Figure 2

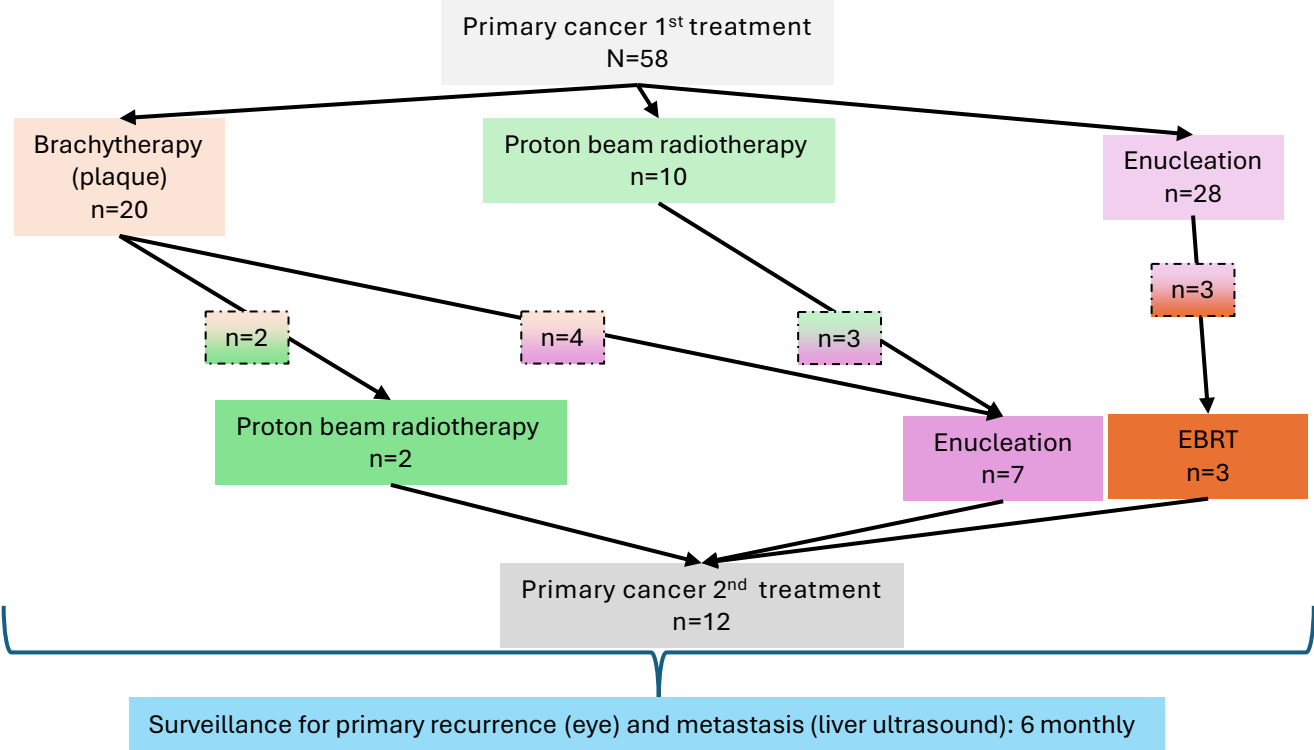

Supplementary Figure 3

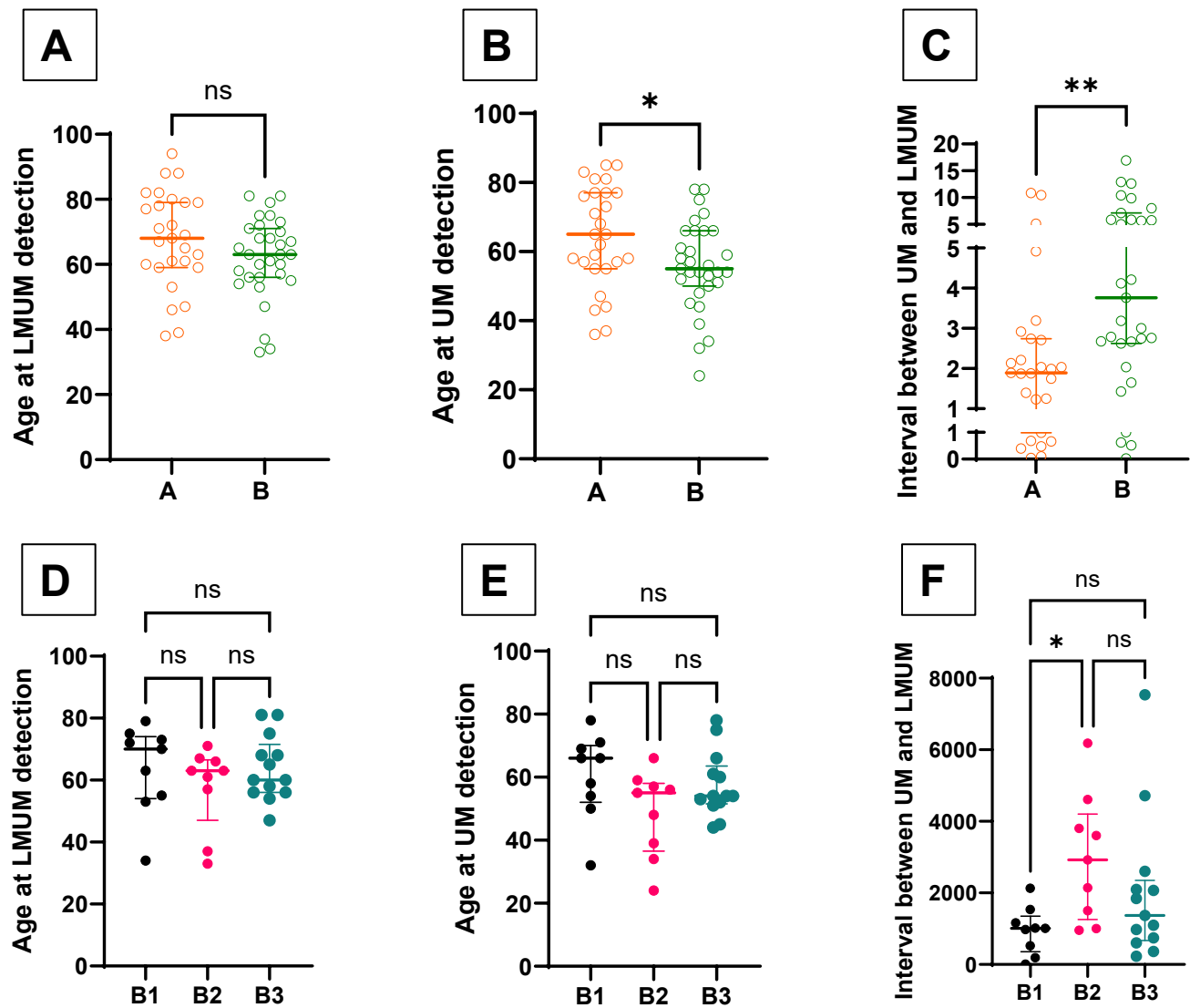

Supplement: Supplementary file 1 [file as9-6-e620-s001.pdf]
